# Two Lip Carcinomas following Allogeneic Hematopoietic Stem Cell Transplantation: A Case Report and Literature Review

**DOI:** 10.1155/2021/6662381

**Published:** 2021-08-27

**Authors:** Nouha Dammak, Latifa Berrezouga, Manel Njima, Ines Lahouel, Mehdi Khemiss, Mohamed Ben Khelifa

**Affiliations:** ^1^Medicine and Oral Surgery Department, University Clinic of Dental Medicine, Monastir, Tunisia; ^2^Dental Medicine Department, Fattouma Bourguiba Teaching Hospital, Monastir, Tunisia; ^3^Anatomical Pathology and Cytology Department, Fattouma Bourguiba Teaching Hospital, Monastir, Tunisia; ^4^Dermatology Department, Fattouma Bourguiba Teaching Hospital, Monastir, Tunisia

## Abstract

**Background:**

Secondary solid cancers are severe complications in patients who have undergone allogeneic hematopoietic stem cell transplantation (AHSCT) for malignant and nonmalignant lymphohematopoietic diseases.

**Objective:**

The aim of this work was to report a case of two lip carcinomas following AHSCT and to warn doctors about the importance of regular check-ups of patients who have received HSCT. *Observation*. A 57-year-old man was referred by the dermatology department for the management of exophytic budding lesions on the lower lip evolving since 5 months. The patient was in complete remission following allogeneic bone marrow transplantation for acute myeloid leukemia since five years. Clinical and histological findings confirmed the diagnosis of a squamous cell carcinoma of the two lesions.

**Conclusion:**

It is of paramount importance to seek an oral squamous cell carcinoma in the presence of persistent lesions in HSCT recipients.

## 1. Introduction

Following advances in hematopoietic stem cell transplantation (HSCT), an increasing number of patients with hematologic malignancies having cured, leading to a steady increase in the long-term survival among recipients. Unfortunately, survivors are developing late complications following HSCT, for example, secondary solid cancers (SSCs), which are the most serious ones with considerable morbidity and mortality. In fact, in a recent study [1] including nearly 2000 AHSCT recipients, the reported cumulative incidence of SSCs was as follows: 3.2% at 5 years, 8.7% at 10 years, and 13.9% at 15 years after transplantation. Indeed, it has been reported that the risk of developing SSC is 2- to 4-fold higher than that in the general population [2], however, according to Shah et al., the risk of developing oral cancer is 14 times higher [3].

It's worth noting that skin and oral cancers account for approximately one-third of all SSCs in HSCT patients, with oral squamous cell carcinoma being predominant with 50% of the cases [4–6]. That is why, since the first published case of oral carcinoma after AHSCT by Lishner et al. [7] in 1990, several cases of secondary oral cancer have been reported in the literature. Thus, the present work describes a male patient who developed lower lip squamous cell carcinomas five years after undergoing AHSCT for the treatment of a previous acute myeloid leukemia (AML). A literature review about the development of oral cancer following HSCT was conducted, as well.

## 2. Case Report

**Figure 1:**
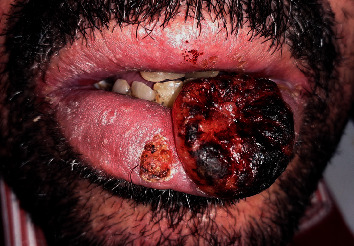
Preoperative view: small lesion with regular round border on the middle lower lip and a large, budding, crusty, and hemorrhagic lesion on the left side of the lower lip.

A 57-year old man was referred in October 2019 by the dermatology department for the investigation of lesions of the lower lip. No history of tobacco or alcohol consumption was recorded. In 2015, the patient underwent AHSCT, following AML with a conditioning regimen including busulfan and cyclophosphamide. A few months later, he developed bronchiolitis obliterans, as an immunological reaction, requiring cyclosporine A and prednisolone as a treatment for a chronic graft versus host disease (cGvHD). The extra oral examination revealed the presence of two concomitant lesions on the lower lip; the first one was small (10 mm) with a regular round border and tender on palpation, the second one was rather budding, large (approximately 40 mm), crusty, and hemorrhagic (Figure 1). According to the patient, the small lesion appeared three weeks ago, while the largest one has evolved since five months. No cervicofacial lymphadenopathy was detected on palpation. The intraoral examination showed a poor oral hygiene and the absence of other mucosa lesions.

**Figure 2:**
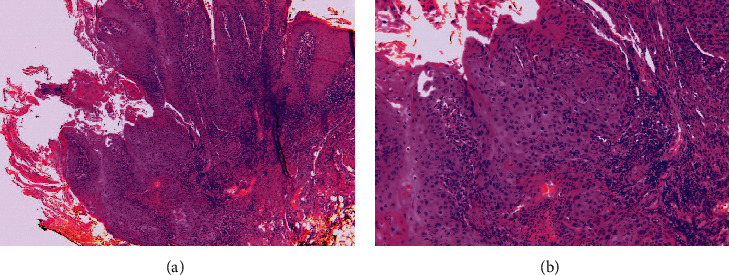
Histological sections: infiltrating carcinomatous proliferation showing squamous differentiation of the labial mucosa (a) small lesion, H&E: 40; (b) large lesion, H&E: 100x.

The incisional biopsy of the two lesions, performed under local anesthesia, concluded to well-differentiated squamous cell carcinomas after histological exams (Figure 2). The work-up for extension carried out by ultrasound images and cervico abdomino pelvic computed tomography failed to show associated lymphadenopathy. Indeed, biological exams and full blood counts were within normal limits. The patient was referred to the maxillofacial surgery department, where complete excision of the lesions was undertaken under local anesthesia. Histological exams of the specimen confirmed the diagnosis of well-differentiated squamous cell carcinomas (T2N0M0). The outcome was favorable after a-year follow-up. No evidence of recurrence or metastasis was recorded. The patient was scheduled for a lip reconstructive surgery.

## 3. Literature Review

A review of the literature was conducted using MEDLINE database via its interface PubMed with the following Mesh terms: “cancer, squamous cell,” “oral cancer,” “carcinoma, squamous cell,” “bone marrow cell transplantation,” and “hematopoietic stem cell transplantation” and combining the following Boolean equations: ((((cancer, squamous cell [MeSH Terms]) OR (oral cancer [MeSH Terms]) OR (oral cavity [MeSH Terms]) OR (carcinoma, squamous cell [MeSH Terms]))) AND ((bone marrow cell transplantation [MeSH Terms]) OR (hematopoietic stem cell transplantation [MeSH Terms])) until August 2020. Selected publications were analyzed according to the following inclusion criteria: HSCT were bone marrow origin, age at OSCC ≥14 years. The exclusion criteria were articles published in a language other than English or French, editor letter, review papers, and conference abstracts. This research concluded to 44 case reports, from 24 articles, about OSCC following HSCT. The information extracted from these cases is summarized in Table 1 and concerned: gender, age at OSCC, tobacco and alcohol use, reason for HSCT, conditioning regimen, interval OSCC-HSCT, GvHD involvement, GvHD treatment or prophylaxis drugs, site and stage of OSCC, and HPV status and outcomes.

## 4. Discussion

Malignant and nonmalignant hematological diseases are widely treated by allogeneic hematopoietic stem cell transplantation (HSCT) from peripheral blood, bone marrow, or cord blood. Although HSCT offers curative therapy for these diseases, it exposes recipient survivors to the risk of secondary malignancies development which are classified into three types: posttransplant lymphoproliferative disorders (PTLD), hematologic malignancies, and solid cancers [5, 9]. The most common secondary malignancies include non-Hodgkin's lymphoma, Hodgkin disease, leukemia in donor-derived cells, and granulocytic sarcoma [27]. Even though, SSCs are less common, a large cohort study [28] conducted by the Center for International Blood and Marrow Transplant Research (CIBMTR) and the Fred Hutchinson Cancer Research Center (FHCRC) reported an increased risk of SSCs. In fact, 189 out of 28.874 included patients developed SSCs after HSCT, with a predominant location in the oral cavity. According to Mawardi et al. [29], oral squamous cell carcinoma (OSCC) is the most common amongst SSCs, with nearly 14 times the risk of developing this cancer compared to the general population. Nevertheless, few case reports and small case series of OSCC following AHSCT have been reported. The present literature review was conducted on oral cancer secondary to HSCT focusing on risk factors related to this condition. Although the conflicting data, several studies have been established to investigate risk factors that might be involved in the development of SSCs in HSCT survivors [2, 28, 30–32].

As for gender, men were considered to be at higher risk for the development of secondary head and neck cancers following HSCT than women [2, 28, 30]. The present review showed 25 males affected and 19 females. Rizzo et al. [28] and Curtis et al. [33] found that males appeared to be slightly higher affected than females, 79% and 72%, respectively. Moreover, the age at transplantation is more likely to be related to cancer development. The mean age at diagnosis of OSCC was 39.61 years old, and 13 patients were aged more than 50 years. According to Gallagher and Forrest [32] and Shimada et al. [31], a great risk of SSCs seems to be associated to an age over 40 and 45 years, respectively.

However, information regarding tobacco and alcohol consumption is lacking, and knowing that these factors are commonly associated with head and neck cancer development, only 6 patients were reported as being addicted to tobacco and alcohol consumption [3, 14, 21, 26]. In fact, Curtis et al. [33] failed to prove that tobacco and alcohol are risk factors in the development of OSCC in HSCT survivors.

Results are controversial regarding the relation between the primary disease and the risk of SSCs. Several studies found higher risk for patients diagnosed with Fanconi anemia (FA) and aplastic anemia (AA) [13]. However, Curtis et al. [30] reported that the risk was rather higher in patients with acute and chronic leukemia. Indeed, Martelin et al. [1] concluded that chronic myeloid leukemia (CML) was an independent risk factor for SSCs. The present review showed a predominance of acute myeloid leukemia (AML) (15 cases) against 6 cases of CML [10, 12, 16, 18, 21] and 5 cases of FA [11, 23, 24, 26]. It's worth to note that myelodysplastic syndrome, PTLD, non-Hodgkin's lymphoma, and acute leukemia predominate early post-HSCT period, while solid cancers occur later [4, 30].

In this review, the incidence of SSCs was reported to peak between 8 and 9 years after HSCT, and the risk reaches its peak in children aged 10 years old at the time of transplantation [13]. The median interval for OSCC development was 8.40 years with a longest period of 22 years [9]. Tanaka et al. [2] concluded that the risk of oral cancer development was elevated during the 5–10 years period. Indeed, Rizzo et al. [28] found that risks tended to rise over time for cancers of the oral cavity, and that long-term survivors had significantly elevated (20 to 30 folds) risks of oral cancer during 10 or more years of follow-up.

In the present case, the male patient underwent HSCT for AML at 52 years old. This finding was in agreement with the study of Curtis et al. [30] who concluded that only patients aged 30 years or older at the time of transplantation had a significantly increased risk of solid cancer.

Another considered major risk factor of OSSC development in HSCT recipients is cGvHD with its chronic inflammation. This condition is an alloimmune process secondary to an immune attack by donor T cells that recognize antigens expressed on normal tissues, including those of the oral cavity [34].

A large cohort study [33] showed that the risk for SCCs increased with the increasing grade of cGvHD, and that patients with severe disease had 10-fold greater risk than those without cGvHD. It was also concluded that long-term cGvHD therapy with azathioprine, particularly when combined with cyclosporine and steroids, was a major risk factor of SCC development. In fact the administration for 24 months or more of these drugs can increase sharply the risk of SSCs by nearly 6 to 8 folds. That is the reason why cGvHD, has been described, by some authors, as a precursor state for a potential development of oral cancer in HSCT recipients [3].

The cGvHD has been reported in 25–40% of HSCT survivors [4, 13]. Clinical manifestations most often affect skin, liver, lungs, gastrointestinal tract, and eyes. Aproximately, 80% of these patients present an oral involvement, comprising lichenoid lesions, atrophy, erythema, xerostomia, and oral pain [4, 13]. In this review, 40 out of 44 patients developed GvHD. Of whom, 27 developed cGvHD of the oral mucosa prior to OSCC diagnosis [4–9, 11–13, 15, 16, 18, 20–23, 25]. The location of GvHD was not precised in 8 patients and not mentioned in 4 patients.

In addition to cGvHD, the use of total body irradiation (TBI) and the intensity of the conditioning regimen are risk factors for secondary malignancies. In this review, conditioning regimens consisted of TBI, chemotherapy, cyclophosphamide combined or not to busulfan, and other drugs [4–6, 8, 12, 14, 16, 19–21]. Curtis et al. [30] reported that the risk of cancer development in recipients who underwent irradiation before HSCT is up to 18.4 times higher than the risk for those who did not undergo irradiation. Indeed, Rizzo et al. [28] found that limited field irradiation (LFI) was associated with an increased risk of SCC of the oral cavity. Majhail et al. [35], however, indicated that the risk of secondary solid malignancies in 4318 HSCT recipients for AML or CML using a high-dose busulphan-cyclophosphamide conditioning regimen are increased even without a prior exposure to LFI or TBI, considering that the absolute risk appeared to be relatively lower. Finally, Shimoni et al. [36] reported that the incidence of secondary malignancies has not decreased in the area of reduced-intensity conditioning and may have even increased when compared with myeloablative conditioning or reduced toxicity conditioning. In the present case, the patient was treated by a combination of busulfan and cyclophosphamide, no irradiation regimen was administered, but information regarding GvHD oral manifestations are lacking.

As for oral location, most of OSCCs were located on the tongue (25 cases/56.81%), then the lip (8 cases/18.18%) and the gingiva (5 cases/11.36%). The buccal mucosa and the floor of the mouth were less affected. For lip locations, 7 cases concerned the lower lip [7, 8, 14, 16, 21], and only one case occurred on the upper lip [6]. It is worth noting that, apart from older age, male gender, and fair skin, sun and UV exposure are amongst risk factors involved in actinic or solar cheilosis, which is a premalignant ulcerative lesion most frequently associated with SCC of the lip that represents up to 25–30% of all oral cancers [37]. In the review, almost all patients experienced GvHD, despite the prophylaxis treatment, in some cases. None of the authors reported a history of sun or UV exposure, but a chronic inflammation related to the immune reaction secondary to GvHD. In fact, it has been reported that tissue damage by diffuse T-cell infiltration in the subepithelial buccal mucosa and in the minor salivary glands may be at the origin of lichen-like changes, ulceration, and erythema. These lesions may predispose to malignant transformation along with immunosuppressive therapy [13, 34]. Consequently, as the patient is working at an industrial paint shop and is not an outdoor laborer (for example, farmer) with prolonged sun exposure to the face, we assume that the oral location of the cGvHD is the lower lip, as reported by some authors [6, 21].

Indeed, to the best of our knowledge, this is the first condition where two synchronous cancers appeared on the same anatomical site. The time interval between the two lesions was nearly 4 months; this is in agreement with that recorded by Montebugnoli et al. [13] who described SCC of the tongue and floor of the mouth four months apart. Nonetheless, it is more likely that the small lesion is an extension of the larger one, because, the epithelium might be infiltrated by tumoral cells as the distance between the 2 lesions is very small (less than 2 mm) and histological features of the 2 specimen were identical, as well.

Finally, some authors evaluated the Human Papilloma Virus (HPV) infection in OSCC after HSCT, as it was proposed that head and neck cancers may be correlated with this oncogenic virus infection. In this review, the isolation of HPV high risk genotypes 16 and 18 was conducted in 16 cases. Of these, 4 were positive and 2 of them were located on the lower lip [21]. Some authors postulated that the reactivation of HPV in these immunocompromised recipients may contribute to cancer development [12, 21, 26].

## 5. Conclusion

Dentists and surgeons should be aware of the following;

- Secondary malignancy development is a potential long-term complication in patients receiving HSCT
- HSCT recipients experiencing cGvHD are at higher risk of developing OSCC
- Careful oral examination and regular evaluation for premalignant lesions and biopsy of any suspicious lesions are required for HSCT recipients, even for a long time after transplantation.

Further studies including a large cohort are needed to better understand the relation between GvHGD and the development of SSCs in HSCT recipients.

## Figures and Tables

**Table 1:** Oral squamous cell carcinoma in hematopoetic stem cell transpantation patients.

Author/year	Gender	Tobacco/alcohol	Primary disease	Conditioning	Age at OSCC	Interval OSCC-HSCT (years)	GvHD location	GvHD prophylaxis/treatment	OSCC site	OSCC stage	HPV infection	Follow up/comment
Present case	M	No	AML	Cy/Bu	57	5	Lungs	Cs/Pr	Lower lip	T2N0M0	NM	No recurrence or metastasis at 1 year
Kibbi et al. [8]	M	NM	LL	NM	28	3	Trunk, OM	Pr/tacrolimus antifungal	Lower lip	T2a	P16 –(IHC)	NM
Tsushima et al. [9]	M	No	AML	TBI/Ct	51	22	OM	Cs/Mtx	Gingiva	NM	NM	No recurrence at 28 months
Katz et al. [6]	M	No	L	TBI	68	10	Skin, OM	NM	Buccal mucosa	T1 M0N1	NM	NM
	M	No	AML	NM	18	9	Lip	NM	Upper lip	T1M0N0	NM	NM
Kano et al. [10]	M	NM	MDS	NM	38	6.4	Yes	NM	Oral cavity	NM	NM	NM
	F	NM	CML	NM	45, 46	12.5, 13.6	Yes	NM	Tongue oral cavity	NM	NM	NM
Torres-Pereira et al. [11]	F	No	FA	Cy	18	10	OM	Cs/Mtx	Tongue	NM	NM	No recurrence at 5 years
	F	No	FA	Cy	28	3	Liver, skin, eyes, OM	Cs/Mtx/ISD	Gingiva Mandibular retromolar area	NM	NM	No recurrence at 3 years
Shah et al. [3]	F	No	NM	NM	67	14	NM	Rituximab/Pr	Tongue	T4aN2bM0	NM	NM
	F	Yes	NM	NM	49	17	NM	CellCept/Pr	Tongue	T1N0M0	NM	NM
	M	No	NM	NM	57	2	NM	Cs/Pr/rituximab	Tongue	Multiple T1 and T2N0	NM	NM
Kakei et al. [5]	M	NM	AML	TBICy	35	2	OM, eyes, lungs	Cs/ Pr	Gingiva	NM	P16 + P53 + IHC	NM
Chung et al. [12]	M	No	AML	NM	53	7.1	OM	ISD	Tongue	NM	NM	Neck lymph node, distant metastasis
	M	No	AML	No Rxt	51	3.5	OM	ISD	Tongue	NM	NM	Neck lymph node
	F	No	AML	TBI	16	9.4	OM	ISD	Tongue	NM	NM	Neck lymph node
	M	No	AML	No Rxt	47	11.8	OM	ISD	Tongue	NM	NM	NM
	F	No	AML	No Rxt	54	2.4	OM	ISD	Tongue	NM	NM	NM
	M	No	CML	No Rxt	53	10.7	OM	ISD	Tongue	NM	NM	NM
	F	No	Myeloma	No Rxt	55	8.2	OM	ISD	Tongue	NM	NM	NM
Montebugnoli et al. [13]	F	No	*β*- thallassemia	NM	26	17	Tongue	Steroids/antimycotic drugs	Tongue mouth floor	pT3N0M0 pT2N0M0	P16 –P16 –	No recurrence at 2 years
Chen et al. [14]	F	No	AA	TBI	50	6.9	Yes	Az/steroids	Tongue		HPV 16 – (IHC)	
	M	Yes	AML	No TBI	50	16.4	Yes	Az/steroids	Tongue		HPV 16 –(IHC)	
	F	No	AML	No TBI	53	9.4	Yes	Az/steroids	Buccal mucosa		HPV 16 –(IHC)	
	M	No	AA	No TBI	42	8.8	Yes	Az/steroids	Lower lip		HPV 16 –(IHC)	
	M	Yes	AML	No TBI	45	5.2	Yes	Az/steroids	Tongue		HPV 16 –(IHC)	
Noguchi et al. [15]	F	NM	Hodgkin's L	Ct	46	5	OM	Cs/Pr	Tongue	T1N0M0	NM	No recurrence at 1 year and 6 months
Hamadah et al. [16]	M	NM	CML	Cy/Bu	25	3.8	Oral mucosa	Cs/Mtx	Lower lip	Grade I SCC	NM	No relapse at 10 years
	M	NM	Myeloma	Cy/TBI	42	10	OM, skin, gut	Cs/Mtx	Lower lip		NM	Died after 2 years
Tomihara et al. [17]	M	NM	ALL	TBI	24	13	NM	NM	Buccal mucosa	well-differentiated SCC	NM	Ipsilateral submandibular lymph node metastasis at 4 months
Byun et al. [18]	F	NM	CML	Cy/Bu	17	5	OM, Skin, liver, eyes, lungs	Cs/Pr	Tongue	T2N0M0, stage II.	HPV + (PCR)EBV +	No recurrence or metastasis at 5 months
Demarosi et al. [4]	F	No	Non Hodgkin's L	Cy/TBI	53	5	OM	Cs and Mtx prophylaxis/ISD for treatment	Gingiva	NM	NM	NM
Girod and Breton [19]	F	No	ALL	Cy/TBI	40	5	Skin	Cs	Tongue	pT2NO	NM	No recurrence or metastasis at 9 months
Szeto et al. [20]	M	No	AML	Bu/Cy/TBI	45	6	OM, skin, liver	Cs and Mtx	Tongue	T3N0M0, stage III	NM	Neck recurrence after 7 months, died 3 months later
	M	No	AML	Bu/Cy	48	4	OM, skin, liver, lungs	Cs and Mtx	Tongue	T2N0M0	NM	Good at 2 months
Zhang et al. [21]	M	Yes Alcohol	CML	Ct/TBI	35	8	OM, skin, gastrointestinal tract,	Topical steroid	Tongue	T3N0M0 (stage III)	HPV–(PCR)	No recurrence at 2 years
	M	Yes	CML	TBI	47	7	OM	Cs/dapsone/steroid	Lower lip	Stage I (T1N0M0).	HPV + 18 (PCR)	NM
	M	Not	AML	TBI	54	5	OM, Lip	Topical steroid	Lower lip	CIS	HPV + 16/18 (PCR)	No recurrence at 2 years
Abdelsayed et al. [22]	M	NM	ALL	TBI	24	2	Skin, OM	NM	Buccal mucosa	CIS	HPV–(PCR)	NM
	M	NM	ALL	Ct/TBI	14	8	Skin, lung	NM	Tongue	Well-differentiated SCC	HPV–(HIS)	
Jansisyanont et al. [23]	F	NM	FA	NM	24	15	OM	No treatment	Tongue	T1N0M0	NM	No recurrence or metastasis at 6 months
Millen et al. [24]	F	NM	FA	Cy/TBI	18	9	Skin, gut, liver	Cs/Az	Buccal mucosa	Moderately well differentiated SCC	NM	Retro orbital extension of tumor. Died 3 months postoperatively
Otsubo et al. [25]	F	NM	AA	Cy/Total Lymphoid Irradiation	20	4	Skin, OM	Mtx/Cs prophylaxis and Pr treatment	Gingiva	T3N0M0 Well- differentiated SCC	NM	No recurrence or metastasis
Bradford et al. [26]	F	Yes	FA	NM	29	10	Yes	Steroids	Tongue	Moderately well- differentiated SCC T4N0M0	Probable	Died 1 month postoperatively
Lishner et al. [7]	M	NM	AA	Cy/TBI	41	6	OM, skin, liver	Pr/Az	Lower lip	moderately to poorly differentiated SCC	HPV 16 and 18- (HIS)	Died

Abbreviations: M, male; F, female; LL, lymphoblastic leukemia; L, lymphoma; AML, acute myeloid leukemia; CML, chronic myeloid leukemia; MDS, myelodysplastic syndrome; FA, Fanconi anemia; AA, aplastic anemia; TBI, total body irradiation; Ct, chemotherapy; Cy, cyclophosphamide; Bu, busulphan; Rxt, radiotherapy; OM, oral mucosa; Pr, prednisone; Cs, cyclosporine A; Az, azathioprine; and Mtx, methotrexate; ISD, Immunosuppressive drugs; NM, not mentioned.

## Data Availability

All the data are available from the corresponding authors upon request.
